# Targeted Next-Generation Sequencing of Congenital Hypothyroidism-Causative Genes Reveals Unexpected Thyroglobulin Gene Variants in Patients with Iodide Transport Defect

**DOI:** 10.3390/ijms23169251

**Published:** 2022-08-17

**Authors:** Carlos Eduardo Bernal Barquero, Romina Celeste Geysels, Virginie Jacques, Gerardo Hernán Carro, Mariano Martín, Victoria Peyret, María Celeste Abregú, Patricia Papendieck, Ana María Masini-Repiso, Frédérique Savagner, Ana Elena Chiesa, Cintia E. Citterio, Juan Pablo Nicola

**Affiliations:** 1 Departamento de Bioquímica Clínica, Facultad de Ciencias Químicas, Universidad Nacional de Córdoba, Cordoba 5000, Argentina; 2 Centro de Investigaciones en Bioquímica Clínica e Inmunología, Consejo Nacional de Investigaciones Científicas y Técnicas (CIBICI-CONICET), Cordoba 5000, Argentina; 3 Laboratoire de Biochimie, Institut Fédératif de Biologie, Le Centre Hospitalier Universitaire de Toulouse, 31300 Toulouse, France; 4 Institut des Maladies Métaboliques et Cardiovasculaires (I2MC), Institut National de la Santé et de la Recherche Médicale (INSERM) UMR 1297, 31432 Toulouse, France; 5 División de Endocrinología, Hospital de Niños Dr. Ricardo Gutiérrez, Buenos Aires 1006, Argentina; 6 Centro de Investigaciones Endocrinológicas Dr. César Bergadá, Consejo Nacional de Investigaciones Científicas y Técnicas (CEDIE-CONICET), Buenos Aires 1120, Argentina; 7 Instituto de Inmunología, Genética y Metabolismo, Consejo Nacional de Investigaciones Científicas y Técnicas (INIGEM-CONIET), Buenos Aires 1120, Argentina; 8 Division of Metabolism, Endocrinology and Diabetes, University of Michigan Medical School, Ann Arbor, MI 48109, USA

**Keywords:** congenital hypothyroidism, thyroid dyshormonogenesis, iodide transport defect, sodium iodide symporter, thyroglobulin

## Abstract

Congenital iodide transport defect is an uncommon autosomal recessive disorder caused by loss-of-function variants in the sodium iodide symporter (NIS)-coding *SLC5A5* gene and leading to dyshormonogenic congenital hypothyroidism. Here, we conducted a targeted next-generation sequencing assessment of congenital hypothyroidism-causative genes in a cohort of nine unrelated pediatric patients suspected of having a congenital iodide transport defect based on the absence of ^99m^Tc-pertechnetate accumulation in a eutopic thyroid gland. Although, unexpectedly, we could not detect pathogenic *SLC5A5* gene variants, we identified two novel compound heterozygous *TG* gene variants (p.Q29* and c.177-2A>C), three novel heterozygous *TG* gene variants (p.F1542Vfs*20, p.Y2563C, and p.S523P), and a novel heterozygous *DUOX2* gene variant (p.E1496Dfs*51). Splicing minigene reporter-based in vitro assays revealed that the variant c.177-2A>C affected normal TG pre-mRNA splicing, leading to the frameshift variant p.T59Sfs*17. The frameshift *TG* variants p.T59Sfs*17 and p.F1542Vfs*20, but not the DUOX2 variant p.E1496Dfs*51, were predicted to undergo nonsense-mediated decay. Moreover, functional in vitro expression assays revealed that the variant p.Y2563C reduced the secretion of the TG protein. Our investigation revealed unexpected findings regarding the genetics of congenital iodide transport defects, supporting the existence of yet to be discovered mechanisms involved in thyroid hormonogenesis.

## 1. Introduction

Congenital hypothyroidism, the most frequent inborn disorder detected in newborn screening programs, is a dysfunction of the hypothalamic-pituitary-thyroid axis that is present at birth, resulting in thyroid hormone deficiency [1]. Although iodine deficiency remains the leading cause of hypothyroidism at birth, in iodide-sufficient areas, over 65% of these patients present abnormalities in thyroid organogenesis (dysgenesis), with the remaining 35% of the patients developing eutopic thyroid glands with impaired thyroid hormonogenesis (dyshormonogenesis) [2]. Recent consensus guidelines from the European Reference Network on Rare Endocrine Conditions recommend molecular diagnosis using next-generation sequencing in order to explore the genetic basis of the disease, as well as facilitating an interdisciplinary follow-up of the patients and guaranteeing the provision of adequate genetic counseling to the families [3].

Current consensus guidelines also recommend that after the identification of the disease in neonatal screening, the biochemical confirmatory diagnosis should be followed by complementary biochemical and imaging studies to obtain a better understanding of the underlying etiology of the disease [3], in particular by using Sanger sequencing-based approaches to evaluate the sequence of candidate genes. The general clinical presentation of congenital iodide transport defect, an autosomal recessive disorder due to impaired iodide accumulation in the thyroid follicular cell, includes a variable degree of hypothyroidism, reduced to absent radioiodide accumulation in a normal to hyperplastic eutopic thyroid gland, a low saliva-to-plasma iodide ratio, and normal to increased thyroglobulin (TG) serum levels [4,5,6,7].

Since iodine is a crucial component of thyroid hormones, iodide accumulation in the thyroid follicular cell constitutes a critical requirement for thyroid hormonogenesis [8]. The sodium/iodide symporter (NIS) is the basolateral plasma membrane glycoprotein involved in the accumulation of iodide into the thyroid follicular cell [9]. The carboxy-terminus of this protein, which is oriented towards the cytoplasm, contains the specific sorting and retention signals required for NIS expression at the basolateral plasma membrane [10,11,12]. Over thirty pathogenic variants in the NIS-coding *SLC5A5* gene have been reported in patients with dyshormonogenic congenital hypothyroidism. The functional characterization of loss-of-function NIS variants identified in patients has provided mechanistic information about the transporter [9]. Given the relevance of NIS in thyroid physiology, we recently developed a machine learning-based NIS-specific variant classifier with the aim of improving the prediction of the pathogenicity of missense NIS variants [13].

Here, using targeted next-generation sequencing, we investigated the presence of pathogenic variants in the *SLC5A5* gene in a cohort of nine pediatric patients with dyshormonogenic congenital hypothyroidism, suspected of being due to an iodide transport defect based on the absence of ^99m^Tc-pertechnetate accumulation in the thyroid gland. Unexpectedly, we could not identify any pathogenic *SLC5A5* gene variants. Therefore, we extended our analysis to a comprehensive panel of 16 other known causative congenital hypothyroidism genes. Surprisingly, we identified two novel compound heterozygous *TG* gene variants (p.Q29* and c.177-2A>C), three novel heterozygous *TG* gene variants (p.F1542Vfs*20, p.Y2563C, and p.S523P), and a novel heterozygous *DUOX2* gene variant (p.E1496Dfs*51). A functional in vitro characterization using splicing minigene reporter assays revealed that the c.177-2A>C variant caused exon 3 skipping during the pre-mRNA splicing process, leading to the TG pathogenic variant p.T59Sfs*17. Moreover, functional in vitro expression assays suggested that the variant p.Y2562C causes a defect in the secretion of TG polypeptides, thus resulting in a reduced export of the protein toward the colloid for thyroid hormonogenesis. Remarkably, the frameshift *TG* variants p.T59Sfs*17 and p.F1542Vfs*20, but not the DUOX2 variant p.E1496Dfs*51, were predicted to undergo degradation by nonsense-mediated decay. In conclusion, our investigation revealed unexpected findings regarding the genetic and phenotypes of congenital iodide transport defects, suggesting the existence of yet to be discovered mechanisms being involved in thyroid hormonogenesis.

## 2. Results

### 2.1. Clinical, Biochemical and Imaging Characteristics of the Study Cohort

The study recruited a cohort of nine patients with permanent congenital hypothyroidism and suspected of iodide transport defect based on non-detectable 9^9m^Tc-pertechnetate uptake in a eutopic non-goitrous thyroid gland (Table 1). All patients were detected through newborn screening for congenital hypothyroidism using TSH levels (Table 1). Confirmatory thyroid function tests showed increased TSH and reduced total and free T4 serum levels. TG serum levels were mostly found to be in the normal range, and all patients tested negative for thyroid autoantibodies (Table 1).

### 2.2. Genetic Analysis of Congenital Hypothyroidism Using Targeted Next-Generation Sequencing

Based on the radionuclide scintigraphy analysis revealing non-detectable 9^9m^Tc-pertechnetate uptake in the thyroid gland, we explored the presence of pathogenic *SLC5A5* gene variants using targeted next-generation sequencing. Unexpectedly, pathogenic *SLC5A5* gene variants were not detected. Interestingly, a radionuclide imaging of the salivary glands revealed an unexpected classification of the patient phenotype into two groups; patients with detectable (thyroid iodide transport defect) or non-detectable (complete iodide transport defect) ^99m^Tc-pertechnetate uptake (Table 1). Patients with congenital iodide transport defects caused by pathogenic *SLC5A5* gene variants do not accumulate iodide either in the thyroid or in the salivary gland, as NIS-mediated iodide transport is impaired [14,15].

Next, we extended our analysis to a comprehensive panel of 16 other known causative congenital hypothyroidism genes. Targeted next-generation sequencing analysis revealed novel compound heterozygous (p.Q29* and c.177-2A>C) and heterozygous (p.F1542Vfs*20) *TG* gene variants in patients with a complete iodide transport defect phenotype (Table 1). Moreover, the sequencing analysis revealed novel heterozygous *TG* gene variants (p.Y2563C and p.S523P) and *DUOX2* gene variants (p.E1496Dfs*51) in patients with a thyroid iodide transport defect phenotype (Table 1).

The variant prioritization pipeline led to the absence of significant variants in any of the sequenced genes in four patients of the study cohort (Table 1). Among these patients, two revealed thyroid iodide transport defects and two were not classified, as no record of ^99m^Tc-pertechnetate scintigraphy in the salivary glands was available.

### 2.3. In Silico Analysis of Variants Identified in Congenital Hypothyroidism-Causative Genes

Although not associated with congenital hypothyroidism, the TG variants p.Q29*, p.Y2563C and p.S523P have been reported in the Single Nucleotide Polymorphism database in heterozygosis, which showed a minor allele frequency of less than 1% (rare variants) according to The Genome Aggregation Database (Table 2). Nonsense (p.Q29* TG) variants and frameshift variants leading to premature stop codons (p.F1542Vfs*20 TG and p.E1496Dfs*51 DUOX2) were considered to be pathogenic. The algorithm NMDEscPredictor [16] predicted that the transcript encoding the frameshift TG variant p.F1542Vfs*20, but not the DUOX2 variant p.E1496Dfs*51, undergoes degradation by nonsense-mediated decay. Moreover, an in silico analysis of missense TG variants using prediction algorithms revealed that p.S523P is benign, whereas p.Y2563C is pathogenic (Table 2).

**Figure 1:**
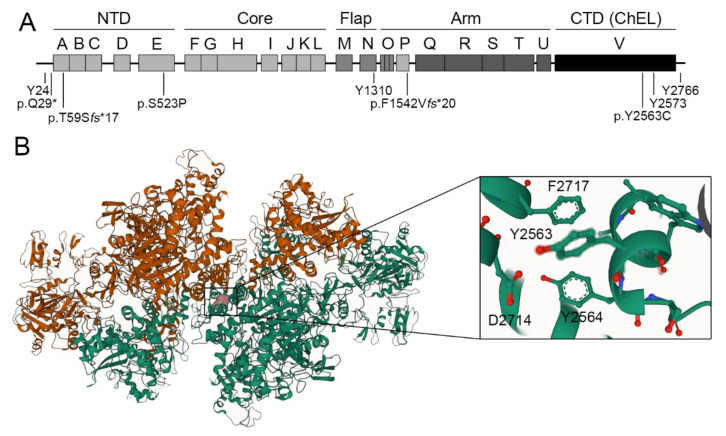
Schematic representation of detected TG variants. (**A**) Representation of TG variants in the primary structure of the human TG monomer. In the context of the 3D structure, the monomer of TG was divided into five regions: the amino-terminal domain (NTD), and the core, flap, arm, and carboxy-terminal domains (CTD), corresponding to the dimeric choline esterase-like domain (ChEL). Primary sequence-based internal homology domains named type 1, 2, and 3 TG-like repeats, as well as the ChEL domain are labeled as A to V. The hormonogenic tyrosines Y24, Y1310, Y2573, and Y2766 are indicated. (**B**) Representation of the residue p.Y2563C (colored in pink) in one monomer of the cryo-electron microscopy structure of TG homodimer (PDB ID 6SCJ). Monomers are displayed in orange and green. Close-up view of the structure showing Y2563-interacting residues Y2564, D2714 and F2717.

All TG variants were mapped in the primary structure of the human TG monomer (Figure 1A). The variant p.Q29* is adjacent to the type 1 TG-like repeat A, where the variant p.T59Sfs*17 (protein product of c.177-2A>C, see below) is located, with the variant p.S523P being located in the linker domain spacing type 1 TG-like repeats D and F (domain E) within the amino-terminal domain (NTD). Recently, the functional characterization of the missense variant p.L571P revealed the relevance of the linker domain in the intracellular trafficking of TG [17]. The variant p.F1542Vfs*20 is located in the type-1 repeat P within the arm domain, while the variant p.Y2563C is located in the homodimerization carboxy-terminal V domain, also known as the choline esterase-like (ChEL) domain. The ChEL domain is known to be determinant for TG intracellular trafficking along the apical secretory pathway to the follicular lumen, where thyroid hormonogenesis occurs [18,19].

Recently, the cryo-electron microscopy 3D structure of the human TG dimer has revealed that the monomers are intertwined, with each monomer being entangled and revolving around the central ChEL dimer that interacts with the Arm and Core domains of the same monomer, and with the NTD of the partner monomer involving the E domain [20,21]. Using the web-based molecular visualization Mol* Viewer platform [22], we observed that the residue Y2563 was located in a short alpha-helix stretch in the ChEL dimer interface, forming a putative non-covalent intramonomeric hydrogen-bond interaction with D2714 and also pi-stacking interactions with Y2564 and F2717 (Figure 1B). The presence of cysteine residue at position 2563 may not only disrupt intramonomeric molecular interactions, but also reorder the complex disulfide bond network, thereby having an effect on the overall folding of the protein.

### 2.4. The Pathogenic Variant c.177-2A>C Impairs Normal TG Pre-Messenger RNA Splicing

**Figure 2:**
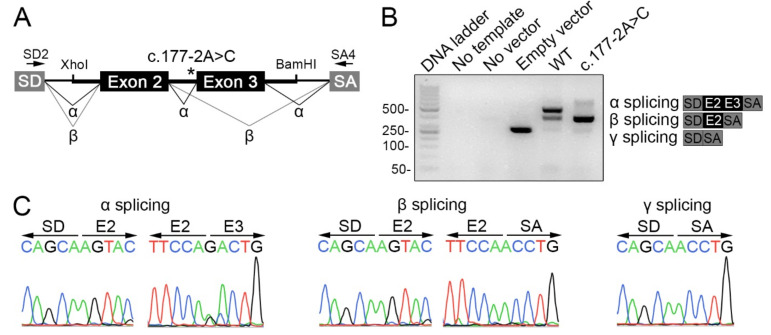
The variant c.177-2A>C causes exon 3 skipping during TG pre-mRNA splicing. (**A**) Scheme of pSPL3-based minigenes used in functional assays. The genomic fragment containing exons 2 (109 bp) and 3 (98 bp) along with a portion of the flanking introns 1 and 3 (247 and 236 bp, respectively), and the spacing intron 2 (1505 bp) was cloned in the multiple cloning site engineered within the single intron spanning splice donor (SD) and acceptor (SA) exons using the XhoI and BamHI restriction sites. Asterisk indicates the position of the variant c.177-2A>C. Arrows show the pSPL3 vector SD and SA exon-specific primers (SD2 and SA4) used in the RT-PCR analysis. Canonical (α) and aberrant (β) splicing products are indicated. (**B**) Agarose gel electrophoresis of RT-PCR products from non-transfected (no vector) and empty, WT or c.177-2A>C pSPL3 minigenes transiently transfected into HeLa cells. In the negative PCR control, cDNA was replaced by nuclease-free water. The empty pSPL3 vector, where only SD-SA exon splicing occurred, led to a 263 bp PCR product (92 bp from exon SD and 171 bp from exon SA) (γ splicing). The WT pSPL3 minigene yielded a 470 bp PCR product including SD-SA exons flanking the exons 2 and 3 of the *TG* gene (α splicing). The c.177-2A>C pSPL3 minigene led to a PCR product of 372 bp including SD-SA exons flanking exon 2 alone (β splicing). The schemes represent the sequence of α, β and γ splicing RT-PCR products. (**C**) Sequencing analysis confirmed the sequence of α, β and γ splicing RT-PCR products.

In silico analysis using splicing prediction algorithms revealed that the variant c.177-2A>C, located in the canonical splicing acceptor site at the boundary of *TG* intron 2 and exon 3, had a deleterious effect on normal *TG* pre-messenger RNA splicing (Table 3). To assess the impact of the variant c.177-2A>C, pSPL3-based minigenes were generated and functionally tested in transiently transfected HeLa cells (Figure 2A). The wild-type (WT) minigene generated a major transcript of 470 bp compatible with the canonical spliced transcript including exons 2 and 3 (α splicing product), and also a minor transcript of 372 bp compatible with a partially spliced transcript including exon 2 alone (β splicing product) (Figure 2B). In contrast, minigene assays revealed that the variant c.177-2A>C generated only one transcript of 372 bp compatible with the skipping of exon 3 (β splicing product). As expected, the empty reporter vector generated a transcript of 263 bp (γ splicing product) (Figure 2B). Sanger sequencing confirmed the identity of all these PCR products (Figure 2C). Significantly, the variant c.177-2A>C changed the open reading frame of the transcript and generated a downstream premature translation stop codon, leading to the TG pathogenic variant p.T59Sfs*17. The algorithm NMDEscPredictor [16] predicted that the transcript encoding the frameshift variant p.T59Sfs*17 is subjected to degradation by nonsense-mediated decay.

### 2.5. The Variant p.Y2563C Reduces TG Secretion

**Figure 3:**
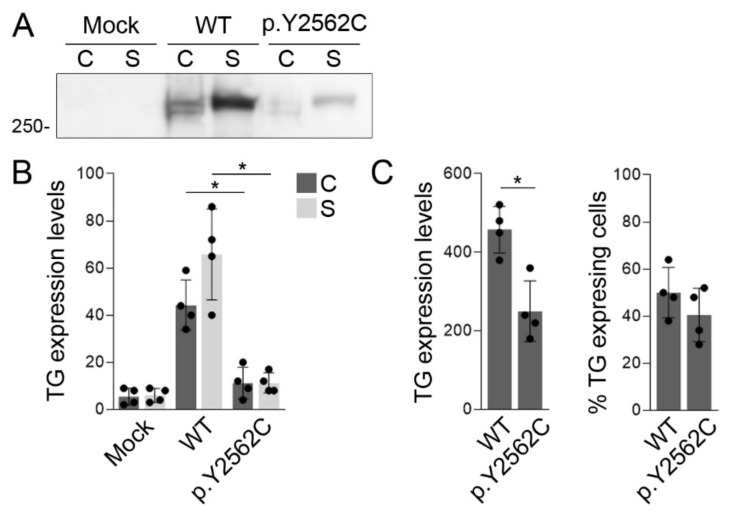
The variant p.Y2563C reduces the secretion of TG polypeptides. (**A**) Representative western blot analysis of TG expression in serum-free supernatants (S) and cell lysates (C) of HEK-293T cells transiently transfected with an empty expression vector (Mock) or expression vectors encoding full-length mouse TG or p.Y2562C TG. Monomers of TG were resolved as a single band of a molecular mass corresponding to ~330 kDa. (**B**) Densitometric analysis of TG expression levels in cell lysates (C) and supernatants (S). Data are expressed as relative units calculated based on the background-subtracted mean intensity of WT or p.Y2562C TG bands ± SD (n = 4). * *p* < 0.05 vs. WT TG-expressing cells (ANOVA, Holm-Sidak tests). (**C**) Flow cytometry analysis to assess transfection efficiency and TG expression levels in permeabilized HEK-293T cells transiently expressing WT or p.Y2562C TG. The TG expression levels are expressed as median fluorescence intensity (MFI) ± SD (n = 4). * *p* < 0.05 vs. WT TG-expressing cells (Student’s *t*-test).

In order to assess the pathogenicity of the TG variant p.Y2563C, we conducted functional assays in the HEK-293T cells, which do not express TG endogenously. HEK-293T cells were transiently transfected with an empty expression vector (Mock) or expression vectors encoding full-length mouse WT or p.Y2562C TG (equivalent to p.Y2563C in human TG). Western blot analysis revealed an expected specific band for TG with a molecular mass corresponding to ~330 kDa both in the cell lysate and in the supernatant, denoting TG expression and secretion, respectively (Figure 3A) [17,23]. In contrast, p.Y2562C TG protein expression in the cell lysate and its secretion to the supernatant was significantly reduced compared to WT TG (Figure 3B). In addition, we conducted a flow cytometry analysis to assess TG expression in permeabilized HEK-293T cells transiently expressing empty vector and WT or p.Y2562C TG. The data analysis showed that, although the percentage of transfection efficiency was comparable between WT and p.Y2562C TG transiently expressing cells, the levels of p.Y2562C TG were significantly lower than those of WT TG (Figure 3C). A putative dominant negative effect of p.Y2562C TG on the secretion of WT TG was dismissed as TG serum levels in patient #3 carrying p.Y2563C TG in the heterozygous state were found to be in the normal range (Table 1). Taken together, our findings suggest that the pathogenic variant p.Y2562C causes a possible defect in the stability of TG polypeptides in HEK-293T cells, thereby leading to a reduced expression and secretion of the protein for thyroid hormonogenesis.

## 3. Discussion

Using targeted next-generation sequencing, we investigated the presence of pathogenic variants in the *SLC5A5* gene in a study cohort of nine unrelated patients with permanent dyshormonogenic congenital hypothyroidism and that were suspected of having an iodide transport defect. Unexpectedly, our analysis did not uncover pathogenic variants in the *SLC5A5* gene to account for the genetic basis of the thyroid disease. Recently, we reported a Sanger sequencing-based study which also assessed the presence of pathogenic *SLC5A5* gene variants in a study cohort of four patients suspected of congenital iodide transport defect, but we only identified the homozygous synonymous c.1326A>C variant, which led to aberrant NIS pre-mRNA splicing in one patient of the study cohort [24]. Although we focused on the coding region and exon-intron boundaries, other potential mechanisms, such as large genomic rearrangements or pathogenic variants that affect regulatory elements, including regulatory and deep intronic regions, should also be considered. Interestingly, two recent reports provided functional evidence that intronic variants (c.970-3C>A and c.970-48G>C) cause exon skipping during NIS pre-mRNA splicing, thus leading to iodide transport defects [25,26].

Over a decade ago, Fukata et al. [27] questioned whether the determination of the saliva-to-plasma radioactive iodide ratio is a requirement for the diagnosis of iodide transport defects. In addition to the thyroid, NIS also mediates active iodide transport in several extra-thyroidal tissues, including the salivary glands, stomach, small intestine, and lacrimal glands [28]. Consequently, in the absence of functional NIS molecules, iodide has no access to the thyroid (hampering thyroid hormonogenesis) or any extra-thyroidal tissue. These authors recommended first confirming the presence of eutopic thyroid glands by neck ultrasonography, and, if present, then conducting radionuclide scintigraphy to explore NIS-mediated iodide accumulation [27]. If neither the thyroid gland nor the salivary glands are observed to show NIS-mediated iodide accumulation during radionuclide scintigraphy, then a screening of pathogenic *SLC5A5* gene variants is encouraged for molecular diagnosis [27]. Here, we uncovered through a radionuclide scintigraphy analysis of the salivary glands an unexpected classification of patients with defective iodide accumulation in the thyroid gland into two groups: patients with detectable (thyroid iodide transport defect) or non-detectable (complete iodide transport defect) ^99m^Tc-pertechnetate accumulation in the salivary glands. Based on these findings, it is reasonable to speculate that pathogenic variants may exist in yet to be discovered thyroid-specific genes, which were not targeted in our sequencing strategy, and are likely to be required for NIS-mediated iodide transport in the thyroid follicular cell. In line with this, the thyroid-stimulating hormone (TSH) is the primary hormonal regulator of thyroid NIS expression [29], and pathogenic variants in the TSH receptor-coding *TSHR* gene have been identified in patients with congenital hypothyroidism showing hypoplastic or normal-sized eutopic thyroid glands with reduced to absent radionuclide uptake on thyroid scintigraphy [30,31]. However, we could not reveal pathogenic variants in the *TSHR* gene in any patient of our cohort with thyroid iodide transport defect.

Targeted next-generation sequencing analysis demonstrated an unexpected enrichment in pathogenic compound heterozygous (p.Q29* and c.177-2A>C) and heterozygous (p.F1542Vfs*20) *TG* gene variants in patients with a complete iodide transport defect phenotype. Moreover, this analysis revealed pathogenic heterozygous *TG* (p.Y2563C) and *DUOX2* (p.E1496Dfs*51) gene variants in some patients with a thyroid iodide transport defect phenotype. Based on the autosomal recessive nature of dyshormonogenic hypothyroidism, despite a discordant correlation between genotype and phenotype, the compound heterozygous variants in the *TG* gene leading to defective TG synthesis may partially explain the etiology of the disease in one of our patients (patient #1). Patients with *TG* defects usually present goiter, moderate to severe hypothyroidism, low to undetectable TG serum levels, and normal to increased iodide accumulation [18]. Unfortunately, TG serum levels were not available in our patient in order to establish further correlation between genotype and phenotype. Although heterozygous pathogenic TG variants are unlikely to explain the defective iodide transport phenotype, they may contribute to modulate the thyroid phenotype in patients with oligogenic forms of congenital hypothyroidism. Structurally defective *TG* variants tend to misfold and accumulate in the endoplasmic reticulum, causing severe thyroidal reticulum stress [32], which could lead to a reduced iodide accumulation and expression of proteins involved in thyroid hormonogenesis, thereby being a possible TSH signaling-dependent counter-regulatory mechanism to reduce endoplasmic reticulum stress [33]. In addition, the absence of *TG* variants in the only patient of our cohort showing reduced TG serum levels (patient #7) does not explain the etiology of the disease. Taking these results together, our analysis reinforces the need to expand the current knowledge on the genetic landscape of the disease.

The advent of next-generation sequencing has expanded the mutational landscape of monogenic forms of congenital hypothyroidism, as well as uncovering the potential oligogenic origin of the disease [2]. Lately, whole-exome sequencing has revealed pathogenic variants in novel genes that lead to thyroid dysgenesis [34,35,36] and dyshormonogenesis [37,38,39]. Although the genetic basis underlying defective iodide accumulation in our study cohort remains elusive, the future implementation of next-generation sequencing-based approaches (i.e., whole-exome or whole-genome sequencing) might reveal novel disease-causing gene variants leading to the complete or thyroid phenotype of defective iodide accumulation. This hypothesis is supported by the observation that the function of the TSH-stimulated KCNQ1/KCNE2 potassium channel is required for thyroid hormonogenesis by sustaining normal NIS-mediated iodide transport in the thyroid follicular cell [40].

## 4. Material and Methods

### 4.1. Patients

Nine unrelated patients with permanent dyshormonogenic congenital hypothyroidism suspected of an iodide transport defect were enrolled in the study. All patients were full-term newborns of non-consanguineous Caucasian (of European descent) parents, and showed an abnormally high thyroid-stimulating hormone (TSH) serum level (cut-off value 15 mIU/L) during the newborn screening program (Table 1). The diagnosis of congenital hypothyroidism was made by assessing increased TSH serum levels, with total or free thyroxine (T4) levels being below the normal range (Table 1). The patients revealed variable clinical signs of hypothyroidism at birth, including jaundice, umbilical hernia, cutis marmorata, puffy dull face, and macroglossia, with delayed bone maturation on a knee X-ray suggesting severe intrauterine hypothyroidism. The inclusion criterion was congenital hypothyroidism with a reduced to absent ^99m^Tc-pertechnetate accumulation in the thyroid gland, as assessed by radionuclide scintigraphy, and with the eutopic thyroid gland being examined by ultrasonography. In addition, the accumulation of ^99m^Tc-pertechnetate in the salivary glands was recorded during thyroid gland scintigraphy. A determination of the saliva-to-plasma iodide ratio was not available. Levothyroxine therapy was started immediately after diagnosis at doses of 10–15 µg/kg per day. All patients were re-evaluated at approximately three years of age, as recommended by consensus guidelines [3], with the discontinuation of levothyroxine treatment for a month revealing permanent hypothyroidism. All parents were clinically and biochemically euthyroid. Parental DNA samples were not available for segregation analysis.

### 4.2. Targeted Next-Generation Sequencing

Genomic DNA was purified from peripheral blood mononuclear cells using the Wizard Genomic DNA Purification Kit (Promega, Madison, WI, USA). Genomic DNA (10 ng/per sample) was used for library preparation, using the DNA prep with an enrichment protocol (Illumina, San Diego, CA, USA) and a custom targeted panel of the 17 congenital hypothyroidism-causative genes TG (NM_003235.4), TPO (NM_000547.5), DUOXA2 (NM_207581.3), DUOX2 (NM_014080.4), SLC5A5 (NM_000453.2), SLC16A2 (NM_006517.4), SLC26A4 (NM_000441.1), TSHR (NM_000369.2), GNAS1 (NM_000516.4), THRB (NM_000461.4), THRA (NM_003250.5), PAX8 (NM_003466.3), NKX2.1 (NM_001079668.2), NKX2.5 (NM_004387.3), FOXE1 (NM_004473.3), IYD (NM_001164694.1), and SECISBP2 (NM_024077.4) [41]. Next-generation sequencing was performed on the NextSeq 550 platform using the Mid Output kit v2.5 (Illumina). Sequencing coverage of 30x reads was considered to be the minimum requirement for a sequence variant to be considered. Sequence data were processed using custom bioinformatic software pipelines to align reads to the HG19 reference genome. All reported variants were explored using public databases, as well as literature searches. All variants were confirmed by Sanger sequencing on a capillary ABI 3500XL sequencer using the BigDye Terminator v3.1 Cycle Sequencing kit (Thermo-Fisher Scientific, Waltham, MA, USA).

### 4.3. Cloning and Site-Directed Mutagenesis

The mouse TG cDNA sequence (NM_009375.2) cloned into the pcDNA3.1 expression vector has been described previously [42]. The variant p.Y2562C in the mouse Tg cDNA was introduced by site-directed mutagenesis using the QuikChange Lightning Site-Directed Mutagenesis kit (Agilent Technologies, Santa Clara, CA) and the mutagenic primers 5′-GTGCTCCAAGGAGTAACACCATACAGCAGCAGC (forward) and 5′-GCTGCTGCTGTATGGTGTTACTCCTTGGAGCAC (reverse).

A human *TG* sequence containing exons 2 (109 nucleotides) and 3 (98 nucleotides) along with the last 247 nucleotides of intron 1, 1505 nucleotides of intron 2, and the first 236 nucleotides of intron 3, was amplified by PCR using the following primers containing XhoI and BamHI restriction sites 5′-CACACTCGAGGCTGCCTGAGACTTGGTGCCTCATG (forward) and 5′-CACAGGATCCGTGGCTCATGGGAGGAACTGGGTAG (reverse). The DNA fragments were cloned into the corresponding cloning sites of the splicing reporter pSPL3 vector [43]. Site-directed mutagenesis was performed by PCR with the mutagenic oligonucleotides 5′-TGTGTCTCCTCCTCCGGACTGTCCAGTGC (forward) and 5′-GCACTGGACAGTCCGGAGGAGGAGACACA (reverse), using Phusion Hot Start II DNA Polymerase (Thermo-Fisher Scientific). Methylated template plasmid was digested with DpnI (Promega, Madison, WI, USA), and the resulting PCR products were transformed into XL1-Blue competent cells [44]. All constructs were sequenced to verify specific nucleotide substitutions (Macrogen, Seoul, South Korea).

### 4.4. Cell Culture and Transfections

Human embryonic kidney (HEK)-293T (CRL-3216) and HeLa cells (CCL-2, American Type Culture Collection, Rockville, MD) were cultured in Dulbecco Modified Eagle’s Medium (Thermo-Fisher Scientific) supplemented with 10% fetal bovine serum (Natocor, Córdoba, Argentina) and antibiotics [45,46]. HEK-293T cells were transiently transfected with 500 ng plasmid/well in 24-well plates using ViaFect transfection reagent (Promega), and HeLa cells were transfected with 2 µg plasmid/well in 6-well plates using Lipofectamine 2000 (Thermo-Fisher Scientific) [47].

### 4.5. Splicing Minigene Reporter Assays

Total RNA was extracted 24 h after transfection with pSPL3-based minigene reporters using the Direct-zol RNA MiniPrep Kit (Zymo Research, Irvine, CA, USA). Complementary DNA synthesis and PCR amplifications were carried out as previously described [48]. The pSPL3-specific primer set was as follows: SD2 5′-GTGAACTGCACTGTGACAAGCTGC and SA4 5′-CACCTGAGGAGTGAATTGGTCG. RT-PCR products were analyzed by agarose gel electrophoresis. The PCR products were gel purified using Wizard SV Gel and the PCR Clean-Up System (Promega), and sequenced to verify the identity of spliced exons (Macrogen).

### 4.6. Western Blot

One day after transfection, the cells were washed and fed with fresh serum-free media. Then, after 24 h, the supernatants were collected and centrifuged to remove cellular debris. The cells were lysed in RIPA buffer containing protease inhibitor cocktail [17], and equal volumes of supernatants and cell lysates were resolved in SDS-PAGE gel under non-reducing conditions, followed by electrotransference to nitrocellulose membranes and immunoblotting, as previously described [23]. Membranes were blocked and incubated with rabbit monoclonal anti-TG primary antibody (ab156008, Abcam, Cambridge, UK) diluted 1:3000 in PBS containing 0.05% Tween-20. After washing, membranes were incubated with HRP-conjugated donkey anti-rabbit secondary antibody (NA934, GE Healthcare, Piscataway, NJ, USA) diluted 1:5000 in PBS containing 0.05% Tween-20. Bands were visualized using the Bio Lumina kit (Kalium Technologies, Quilmes, Argentina), and images were recorded using a GeneGnome XRQ chemiluminescence imaging system (Syngene Synoptics, Cambridge, UK).

### 4.7. Flow Cytometry

Transfected cells were fixed in 2% paraformaldehyde, permeabilized, and stained with rabbit polyclonal anti-TG primary antibody [49] diluted 1:500 in PBS containing 0.2% human serum albumin and 0.2% saponin [15]. After washing, cells were incubated with Alexa-488-conjugated goat anti-rabbit antibody (A-11008, Molecular Probes, Eugene, OR, USA) diluted 1:250 in PBS containing 0.2% human serum albumin and 0.2% saponin. The fluorescence of ~5 × 10^4^ events per tube was quantitated in a BD FACSCanto II Flow Cytometer (BD Biosciences, San Jose, CA, USA). Data analysis was performed with FlowJo software (Tree Star, Ashland, OR, USA).

### 4.8. Statistical Analysis

Results are presented as the mean ± SEM of at least three independent experiments. Statistical tests were performed using Prism 5.0 software (GraphPad Software, La Jolla, CA, USA). Multiple group analysis was conducted by a one-way ANOVA followed by Holm-Sidaks multiple-comparison post hoc tests. Comparisons between two groups were carried out using the unpaired Student’s *t*-test. Differences were considered significant at *p* < 0.05.

## Figures and Tables

**Table 1:** Summary of biochemical, imaging, and molecular findings.

Patient	1	2	3	4	5	6	7	8	9
Neonatal screening									
Age (days)	20	3	30	6	20	2	30	3	15
TSH (<10 mU/L)	>200	190	>100	157	>100	>100	>100	>100	>100
Biochemical analysis									
Age (days)	37	15	50	27	22	12	43	15	21
TSH (0.8–7.8 μg/dL)	>200	193	>100	372	>100	290	>100	170	>100
T_4_ (6–16.5 μg/dL)	2.6	0.4	0.4	1.4	0.4	1.6	0.4	2.8	3.5
Free T_4_ (1–2.1 ng/dL)	-	0.3	-	0.1	0.1	0.1	0.1	0.2	0.4
T_3_ (100–310 ng/dL)	-	73	32	35	19	57	-	94	-
TG (6–83 ng/mL)	-	65	50	12	60	14	1.8	46	22
Anti-TPO/TG antibodies	Nd	Nd	Nd	Nd	Nd	Nd	Nd	Nd	Nd
Imaging studies									
Ultrasonography	Eu	Eu	Eu	Eu	Eu	Eu	Eu	Eu	Eu
^99m^Tc-pertechnetate scintigraphy (thyroid)	Nd	Nd	Nd	Nd	Nd	Nd	Nd	Nd	Nd
^99m^Tc-pertechnetate scintigraphy (salivary glands)	Nd	Nd	Po	Po	Po	-	Po	Po	-
Molecular diagnosis									
Gene variant	*TG* p.Q29*/ c.177-2A>C	*TG* p.F1542Vfs*20	*TG* p.Y2563C	*DUOX2* p.E1496Dfs*51	*TG* p.S523P				

Abbreviations: Eu: Euthyroid. Po: Positive. Nd: Non-detectable. (-): Data not available.

**Table 2:** Overview of genetic variants and in silico analysis of missense variants.

Gene	Nucleotide Variant	Protein Variant	SNP	Allele Frequency gnomAD	In Silico Analysis			ACMG Guideline
					SIFT	PolyPhen-2	MetaLR	
*TG*	c.85C>T	p.Q29*	rs1554648860	0.0000007				PVS1, PM2, PM3, PM4 (Pathogenic)
*TG*	c.177-2A>C	p.T59Sfs*17						PVS1, PS3, PM2, PM3, PP3 (Pathogenic)
*TG*	c.4623_4624insG	p.F1542Vfs*20						PVS1, PM2 (Pathogenic)
*TG*	c.7688A>G	p.Y2563C	rs368587657	0.0000318	0	0.995	0.586	PM2, PP3 (Uncertain significance)
*TG*	c.1567T>C	p.S523P	rs116062097	0.001536	0.06	0.026	0.222	BS1, BP4 (Likely benign)
*DUOX2*	c.4487_4488insC	p.E1496Dfs*51						PVS1, PM2 (Pathogenic)

In silico predictions were carried out using SIFT (score: 1 = tolerated, 0 = deleterious), PolyPhen-2 (score: 0 = benign, 1 = probably damaging), and MetaLR (score: 0 = tolerated, 1 = damaging). Pathogenic scores are indicated in bold. Abbreviations: SNP, Single Nucleotide Polymorphism. gnomAD, Genome Aggregation Database. SIFT, Sorting Intolerant from Tolerant. Polyphen-2, Polymorphism Phenotypic version 2. ACMG, American College of Medical Genetics.

**Table 3:** In silico analysis of the variant c.177-2A>C *TG*.

Variant	Sequence	NNSplice (0–1)	HSF (0–100)	MES (0–16)	ASSP ≥4.5
WT	cctcagGAC	0.93	94.22	9.4	9.878
c.177-2A>C	cctccgGAC	0 (deleterious)	65.28 (deleterious)	1.36 (deleterious)	0 (deleterious)

The variant c.177-2A>C, located at position -2 in the splice acceptor site of intron 2 (lowercase) of the TG gene, is underlined. The exon 3 sequence is shown in capital letters. Scores obtained for WT and the c.177-2A>C splice acceptor site using the indicated software are shown. For each software, the scale range is indicated in parentheses. A deleterious effect of the variant is predicted if the change in variation is greater than 15% relative to WT (% variation = [(variant score—WT score)/WT score] × 100). Abbreviations: NNSplice, Splice Site Prediction by Neural Network. HSF, Human Splicing Finder. MES, MaxEntScan. ASSP, Alternative Splice Site Predictor.

## Data Availability

Data sharing is not applicable to this article.

## References

[1] Nicola J.P. (2017). Clinical Relevance of Molecular Diagnosis in Patients with Congenital Hypothyroidism. J. Mol. Genet. Med..

[2] Stoupa A., Kariyawasam D., Muzza M., de Filippis T., Fugazzola L., Polak M., Persani L., Carre A. (2021). New genetics in congenital hypothyroidism. Endocrine.

[3] Van Trotsenburg P., Stoupa A., Leger J., Rohrer T., Peters C., Fugazzola L., Cassio A., Heinrichs C., Beauloye V., Pohlenz J. (2021). Congenital hypothyroidism: A 2020–2021 consensus guidelines update—An ENDO-European Reference Network initiative endorsed by the European Society for Pediatric Endocrinology and the European Society for Endocrinology. Thyroid.

[4] Zhang C.X., Zhang J.X., Yang L., Zhang C.R., Cheng F., Zhang R.J., Fang Y., Wang Z., Wu F.Y., Li P.Z. (2021). Novel Compound Heterozygous Pathogenic Mutations of SLC5A5 in a Chinese Patient with Congenital Hypothyroidism. Front. Endocrinol..

[5] Durgia H., Nicholas A.K., Schoenmakers E., Dickens J.A., Halanaik D., Sahoo J., Kamalanathan S., Schoenmakers N. (2022). Brief Report: A Novel Sodium/Iodide Symporter Mutation, S356F, Causing Congenital Hypothyroidism. Thyroid.

[6] Nicola J.P., Nazar M., Serrano-Nascimento C., Goulart-Silva F., Sobrero G., Testa G., Nunes M.T., Munoz L., Miras M., Masini-Repiso A.M. (2011). Iodide transport defect: Functional characterization of a novel mutation in the Na+/I− symporter 5′-untranslated region in a patient with congenital hypothyroidism. J. Clin. Endocrinol. Metab..

[7] Nicola J.P., Reyna-Neyra A., Saenger P., Rodriguez-Buritica D.F., Gamez Godoy J.D., Muzumdar R., Amzel L.M., Carrasco N. (2015). Sodium/Iodide Symporter Mutant V270E Causes Stunted Growth but No Cognitive Deficiency. J. Clin. Endocrinol. Metab..

[8] Martín M., Geysels R.C., Peyret V., Bernal Barquero C.E., Masini-Repiso A.M., Nicola J.P. (2019). Implications of Na+/I− Symporter Transport to the Plasma Membrane for Thyroid Hormonogenesis and Radioiodide Therapy. J. Endoc. Soc..

[9] Ravera S., Reyna-Neyra A., Ferrandino G., Amzel L.M., Carrasco N. (2017). The Sodium/Iodide Symporter (NIS): Molecular Physiology and Preclinical and Clinical Applications. Annu. Rev. Physiol..

[10] Martin M., Modenutti C.P., Peyret V., Geysels R.C., Darrouzet E., Pourcher T., Masini-Repiso A.M., Marti M.A., Carrasco N., Nicola J.P. (2019). A Carboxy-Terminal Monoleucine-Based Motif Participates in the Basolateral Targeting of the Na+/I− Symporter. Endocrinology.

[11] Martín M., Salleron L., Peyret V., Geysels R.C., Darrouzet E., Lindenthal S., Bernal-Barquero C.E., Masini-Repiso A.M., Pourcher T. (2021). The PDZ protein SCRIB regulates sodium/iodide symporter (NIS) expression at the basolateral plasma membrane. FASEB J..

[12] Martín M., Modenutti C.P., Gil Rosas M.L., Peyret V., Geysels R.C., Bernal Barquero C.E., Sobrero G., Muñoz L., Signorino M., Testa G. (2021). A Novel SLC5A5 Variant Reveals the Crucial Role of Kinesin Light Chain 2 in Thyroid Hormonogenesis. J. Clin. Endocrinol. Metab..

[13] Martín M., Nicola J.P. (2021). Impact of the mutational landscape of the sodium/iodide symporter in congenital hypothyroidism. Thyroid.

[14] Nicola J.P., Carrasco N., Amzel L.M. (2014). Physiological sodium concentrations enhance the iodide affinity of the Na+/I− symporter. Nat. Commun..

[15] Ferrandino G., Nicola J.P., Sanchez Y.E., Echeverria I., Liu Y., Amzel L.M., Carrasco N. (2016). Na+ coordination at the Na2 site of the Na+/I− symporter. Proc. Natl. Acad. Sci. USA.

[16] Coban-Akdemir Z., White J.J., Song X., Jhangiani S.N., Fatih J.M., Gambin T., Bayram Y., Chinn I.K., Karaca E., Punetha J. (2018). Identifying Genes Whose Mutant Transcripts Cause Dominant Disease Traits by Potential Gain-of-Function Alleles. Am. J. Hum. Genet..

[17] Citterio C.E., Siffo S., Moya C.M., Pio M.G., Molina M.F., Scheps K.G., Rey O.A., Arvan P., Rivolta C.M., Targovnik H.M. (2020). p. L571P in the linker domain of rat thyroglobulin causes intracellular retention. Mol. Cell Endocrinol..

[18] Citterio C.E., Rivolta C.M., Targovnik H.M. (2021). Structure and genetic variants of thyroglobulin: Pathophysiological implications. Mol. Cell Endocrinol..

[19] Lee J., di Jeso B., Arvan P. (2008). The cholinesterase-like domain of thyroglobulin functions as an intramolecular chaperone. J. Clin. Investig..

[20] Coscia F., Taler-Vercic A., Chang V.T., Sinn L., O’Reilly F.J., Izore T., Renko M., Berger I., Rappsilber J., Turk D. (2020). The structure of human thyroglobulin. Nature.

[21] Adaixo R., Steiner E.M., Righetto R.D., Schmidt A., Stahlberg H., Taylor N.M.I. (2022). Cryo-EM structure of native human thyroglobulin. Nat. Commun..

[22] Sehnal D., Bittrich S., Deshpande M., Svobodova R., Berka K., Bazgier V., Velankar S., Burley S.K., Koca J., Rose A.S. (2021). Mol* Viewer: Modern web app for 3D visualization and analysis of large biomolecular structures. Nucleic Acids Res..

[23] Zhou W., Brumpton B., Kabil O., Gudmundsson J., Thorleifsson G., Weinstock J., Zawistowski M., Nielsen J.B., Chaker L., Medici M. (2020). GWAS of thyroid stimulating hormone highlights pleiotropic effects and inverse association with thyroid cancer. Nat. Commun..

[24] Geysels R.C., Bernal Barquero C.E., Martin M., Peyret V., Nocent M., Sobrero G., Munoz L., Signorino M., Testa G., Castro R.B. (2022). Silent but Not Harmless: A Synonymous SLC5A5 Gene Variant Leading to Dyshormonogenic Congenital Hypothyroidism. Front. Endocrinol..

[25] Bernal Barquero C.E., Martín M., Geysels R.C., Peyret V., Papendieck P., Masini-Repiso A.M., Chiesa A.E., Nicola J.P. (2022). An intramolecular ionic interaction linking defective sodium/iodide symporter transport to the plasma membrane and dyshormonogenic congenital hypothyroidism. Thyroid.

[26] Albader N., Zou M., BinEssa H.A., Abdi S., Al-Enezi A.F., Meyer B.F., Alzahrani A.S., Shi Y. (2022). Insights of Noncanonical Splice-site Variants on RNA Splicing in Patients with Congenital Hypothyroidism. J. Clin. Endocrinol. Metab..

[27] Fukata S., Hishinuma A., Nakatake N., Tajiri J. (2010). Diagnosis of iodide transport defect: Do we need to measure the saliva/serum radioactive iodide ratio to diagnose iodide transport defect?. Thyroid.

[28] Jhiang S.M., Sipos J.A. (2021). Na+/I− symporter expression, function, and regulation in non-thyroidal tissues and impact on thyroid cancer therapy. Endocr. Relat. Cancer.

[29] Riesco-Eizaguirre G., Santisteban P., de la Vieja A. (2021). The complex regulation of NIS expression and activity in thyroid and extrathyroidal tissues. Endocr. Relat. Cancer.

[30] Camats N., Baz-Redon N., Fernandez-Cancio M., Clemente M., Campos-Martorell A., Jaimes N., Antolin M., Garcia-Arumi E., Blasco-Perez L., Paramonov I. (2021). Phenotypic Variability of Patients with PAX8 Variants Presenting with Congenital Hypothyroidism and Eutopic Thyroid. J. Clin. Endocrinol. Metab..

[31] Labadi A., Grassi E.S., Gellen B., Kleinau G., Biebermann H., Ruzsa B., Gelmini G., Rideg O., Miseta A., Kovacs G.L. (2015). Loss-of-Function Variants in a Hungarian Cohort Reveal Structural Insights on TSH Receptor Maturation and Signaling. J. Clin. Endocrinol. Metab..

[32] Citterio C.E., Targovnik H.M., Arvan P. (2019). The role of thyroglobulin in thyroid hormonogenesis. Nat. Rev. Endocrinol..

[33] Wen G., Ringseis R., Eder K. (2017). Endoplasmic reticulum stress inhibits expression of genes involved in thyroid hormone synthesis and their key transcriptional regulators in FRTL-5 thyrocytes. PLoS ONE..

[34] Yang R.M., Zhan M., Zhou Q.Y., Ye X.P., Wu F.Y., Dong M., Sun F., Fang Y., Zhang R.J., Zhang C.R. (2021). Upregulation of GBP1 in thyroid primordium is required for developmental thyroid morphogenesis. Genet. Med..

[35] Stoupa A., Adam F., Kariyawasam D., Strassel C., Gawade S., Szinnai G., Kauskot A., Lasne D., Janke C., Natarajan K. (2018). TUBB1 mutations cause thyroid dysgenesis associated with abnormal platelet physiology. EMBO Mol. Med..

[36] Carre A., Stoupa A., Kariyawasam D., Gueriouz M., Ramond C., Monus T., Leger J., Gaujoux S., Sebag F., Glaser N. (2017). Mutations in BOREALIN cause thyroid dysgenesis. Hum. Mol. Genet..

[37] Ishii J., Suzuki A., Kimura T., Tateyama M., Tanaka T., Yazawa T., Arimasu Y., Chen I.S., Aoyama K., Kubo Y. (2019). Congenital goitrous hypothyroidism is caused by dysfunction of the iodide transporter SLC26A7. Commun Biol..

[38] Zou M., Alzahrani A.S., Al-Odaib A., Alqahtani M.A., Babiker O., Al-Rijjal R.A., BinEssa H.A., Kattan W.E., Al-Enezi A.F., Al Qarni A. (2018). Molecular Analysis of Congenital Hypothyroidism in Saudi Arabia: SLC26A7 Mutation Is a Novel Defect in Thyroid Dyshormonogenesis. J. Clin. Endocrinol. Metab..

[39] Cangul H., Liao X.H., Schoenmakers E., Kero J., Barone S., Srichomkwun P., Iwayama H., Serra E.G., Salam H., Eren E. (2018). Homozygous loss-of-function mutations in SLC26A7 cause goitrous congenital hypothyroidism. JCI Insight.

[40] Purtell K., Paroder-Belenitsky M., Reyna-Neyra A., Nicola J.P., Koba W., Fine E., Carrasco N., Abbott G.W. (2012). The KCNQ1-KCNE2 K(+) channel is required for adequate thyroid I(−) uptake. FASEB J..

[41] Oliver-Petit I., Edouard T., Jacques V., Bournez M., Cartault A., Grunenwald S., Savagner F. (2021). Next-Generation Sequencing Analysis Reveals Frequent Familial Origin and Oligogenism in Congenital Hypothyroidism with Dyshormonogenesis. Front. Endocrinol..

[42] Citterio C.E., Morishita Y., Dakka N., Veluswamy B., Arvan P. (2018). Relationship between the dimerization of thyroglobulin and its ability to form triiodothyronine. J. Biol. Chem..

[43] Steffensen A.Y., Dandanell M., Jonson L., Ejlertsen B., Gerdes A.M., Nielsen F.C., van Overeem Hansen T. (2014). Functional characterization of BRCA1 gene variants by mini-gene splicing assay. Eur. J. Hum. Genet..

[44] Geysels R.C., Peyret V., Martín M., Nazar M., Reale C., Bernal Barquero C.E., Miranda L., Martí M.A., Vito P., Masini-Repiso A.M. (2021). The transcription factor NF-κB mediates thyrotropin-stimulated expression of thyroid differentiation markers. Thyroid.

[45] Martin M., Bernal Barquero C.E., Geysels R.C., Papendieck P., Peyret V., Masini-Repiso A.M., Chiesa A.E., Nicola J.P. (2019). Novel Sodium/Iodide Symporter Compound Heterozygous Pathogenic Variants Causing Dyshormonogenic Congenital Hypothyroidism. Thyroid.

[46] Di Giusto P., Martin M., Funes Chaban M., Sampieri L., Nicola J.P., Alvarez C. (2022). Transcription Factor CREB3L1 Regulates the Expression of the Sodium/Iodide Symporter (NIS) in Rat Thyroid Follicular Cells. Cells.

[47] Montesinos M.M., Nicola J.P., Nazar M., Peyret V., Lucero A.M., Pellizas C.G., Masini-Repiso A.M. (2016). Nitric oxide-repressed Forkhead factor FoxE1 expression is involved in the inhibition of TSH-induced thyroid peroxidase levels. Mol. Cell Endocrinol..

[48] Stempin C.C., Getsels R.C., Park S., Palacios L.M., Volpini X., Motrán C.C., Acosta Rodríguez E.V., Nicola J.P., Cheng S.Y., Pellizas C.G. (2021). Secreted Factors by Anaplastic Thyroid Cancer Cells Induce Tumor-Promoting M2-Like Macrophage Polarization through a TIM3-Dependent Mechanism. Cancers.

[49] Kim P.S., Arvan P. (1991). Folding and assembly of newly synthesized thyroglobulin occurs in a pre-Golgi compartment. J. Biol. Chem..

